# Establishment of a Bovine Viral Diarrhea Virus Type 2 Intranasal Challenge Model for Assessing Vaccine Efficacy

**DOI:** 10.3389/fvets.2018.00024

**Published:** 2018-02-27

**Authors:** Rebecca Strong, Simon P. Graham, S. A. La Rocca, Rudiger Raue, Ilse Vangeel, Falko Steinbach

**Affiliations:** ^1^Virology Department, Animal and Plant Health Agency, Addlestone, United Kingdom; ^2^Veterinary Medicine Research & Development, Zoetis, Belgium

**Keywords:** bovine viral diarrhea virus type 2, experimental infection, pathogenesis, virus persistence, challenge model

## Abstract

The objective of this study was to develop a bovine viral diarrhea virus type 2 (BVDV-2) challenge model suitable for evaluation of efficacy of BVDV vaccines; a model that mimics natural infection and induces clear leukopenia and viremia. Clinical, hematological and virological parameters were evaluated after infection of two age groups of calves (3 and 9 months) with two BVDV-2 strains (1362727 and 502643). Calves became pyrexic between 8 and 9 days post inoculation and exhibited symptoms, such as nasal discharge, mild depression, cough, and inappetence. Leukopenia with associated lymphopenia and neutropenia was evident in all groups with lowest leukocyte and lymphocyte counts reached 8 dpi and granulocyte counts between 11 and 16 dpi, dependent on the strain and age of the calves. A more severe thrombocytopenia was seen in those animals inoculated with strain 1362727. Leukocyte and nasal swab samples were positive by virus isolation, as early as 3 dpi and 2 dpi respectively, independent of the inocula used. All calves seroconverted with high levels of BVDV-2 neutralizing antibodies. BVDV RNA was evident as late as 90 dpi and provides the first evidence of the presence of replicating virus long after recovery from BVDV-2 experimental infection. In summary, moderate disease can be induced after experimental infection of calves with a low titer of virulent BVDV-2, with leukopenia, thrombocytopenia, viremia, and virus shedding. These strains represent an attractive model to assess the protective efficacy of existing and new vaccines against BVDV-2.

## Introduction

Bovine viral diarrhea virus (BVDV) is a major pathogen of cattle and also infects a diverse range of ruminants worldwide (1). It is a member of the family *Flaviviridae*, genus pestivirus that also includes border disease virus, classical swine fever virus, and atypical pestiviruses. BVDV exists as two genotypes; BVDV-1 and -2, with a tentative third genotype, atypical bovine pestiviruses (2). BVDV-1 and BVDV-2 have been further subdivided into sub-genotypes based on their genetic characteristics (3).

Bovine viral diarrhea virus infection in cattle causes a variety of clinical representations, from an asymptomatic infection to fatal disease (4). Typically, acute infection in postnatal calves causes a mild transient disease lasting 10–14 days consisting of diarrhea, inappetence, respiratory complications, and an overall loss of condition. In addition, a mild pyrexia and transient leukopenia are apparent for 2–3 days with seroconversion usually before 21 days post-infection (dpi). Infectious virus can be detected in blood and bodily secretions from calves as early as 4 dpi and up to 15 days. BVDV RNA has been detected by sensitive RT-PCR techniques long after the acute infection has cleared. This has raised questions about the virus-free status of animals that have cleared the initial infection and raised an antibody response (5–7). It has also been reported to reduce milk yields and impact on reproductive performance. Acute BVDV infection of in-dam heifers can give rise to the birth of persistently infected (PI) calves, which are able to shed large quantities of virus and, thus, infect susceptible herd mates. The majority of BVD control/eradication programs aim to identify these animals through the use of appropriate diagnostic assays and remove them from herds. This in conjunction with vaccination schedules reduces horizontal transmission to naïve herd mates and prevents vertical transmission to the fetus. Therefore, it is necessary to ensure that BVD vaccines afford protection to circulating strains of BVDV-1 and -2.

BVDV-2 strains were first associated with a severe hemorrhagic disease with high mortality in cattle in Canada and USA but strains causing milder disease have been detected subsequently (8, 9). Over the past 10 years, BVDV-2 has been introduced to Europe through the importation of animals and contaminated products. Recent outbreaks have been reported in Germany (10), Poland (11), and Spain (12). A range of pathogenicity of strains has been reported for both BVDV-2 and -1 (13–15). Dose and virulence of strains, together with the age and immunocompetence of the naïve animal have all been implicated as having an effect on the pathogenicity of disease. Indeed recent studies have found that a more severe clinical disease was observed if the calf either received a high dose of BVDV-1, or was immunocompromised by the use of dexamethasone or was only 3 months old (7).

In this study, we aimed to establish an intranasal BVDV-2 challenge model that would allow the study of pathogenicity of disease and evaluation of efficacy of BVDV vaccines without causing severe clinical disease or mortality in naïve animals. If a potential challenge strain caused a mild disease in the field, experimentally it may fail to provide clear and measurable outcomes of disease, such as pyrexia, thrombocytopenia, and leukopenia. However, a highly virulent field strain may generate these markers of disease but the humane end-point may be reached too soon for the efficacy of a vaccine to be fully evaluated. Thus, using two BVDV-2 strains, the progression of disease was monitored using an extensive clinical scoring system, virus isolation techniques, and by studying immunological parameters. Evidence of the presence of viral RNA persisting long after recovery from BVDV-2 infection was additionally evaluated.

## Materials and Methods

### Viruses

The non-cytopathic BVDV-2 strain, 502643, was the first case of BVDV-2 isolated in the UK from a bull suffering from ill-thrift that subsequently died (16). The BVDV-2 strain, 1362727, was isolated from a UK farm in 2006 where the cattle exhibited severe clinical symptoms with associated death in many cases (17). Virus strains were propagated four passages on fetal bovine turbinate (fBT) cells. After 5 days incubation, virus was harvested and titred on fBT cells (16).

### Study Design

Experiments were carried out on clinically healthy calves aged 11–13 weeks (Danish Holstein; male and female calves) or 9 months old (Danish Holstein or Danish Red; female calves). The calves were confirmed as seronegative for antibodies against BVDV (HerdChek^®^ BVDV Antibody ELISA, IDEXX Laboratories, Wetherby, UK) and tested negative in a BVDV-specific RT-PCR (Penrith, APHA). Six calves of 11–13 weeks old and five calves of 9 months old were inoculated intranasally using intranasal applicator with 10^5.0^ TCID_50_ of BVDV-2 strain 1362727 (groups 1A and 1B, respectively). Two groups of five calves (11–13 weeks old and 9 months old) were inoculated intranasally with 10^4.8^ TCID_50_ of BVDV-2 strain 502643 (groups 2A and 2B, respectively). The different inocula were administered in a 2 ml volume with 1 ml delivered per nostril. The inoculation dose was confirmed through back titration of the inocula. Animals were monitored once daily from 2 days prior to inoculation, until 21 dpi for clinical signs and rectal temperatures were recorded. During these clinical observations, calves were scored for nasal discharge, ocular discharge/conjunctivitis, cough, dyspnea, depression, appetite, diarrhea, and dehydration each on a scale from 0 to 3 (absent, mild, moderate, or severe) according to the scoring system used previously (7). The 3-month-old animals inoculated with the BVDV-2 strains (group 1A and 2A) were maintained to assess long-term infection while the group 1B and 2B animals were euthanized at 21 dpi. One or two animals from the group 1A and 2A calves were euthanized for post-mortem examinations at 28, 55, and 90 days post inoculation.

### Ethics Statement

The animal study was conducted at Contract Research Unit of the Royal Veterinary College under Home Office License number PPL 70/6459 Protocol 19b5 and approved by the Pfizer Animal Health Animal Welfare Committee.

### Virus Isolation

Nasal swabs and heparinized blood samples were collected from all animals daily from 0 to 14 dpi and on 16 dpi. To isolate virus from leukocytes, blood was centrifuged at 1500 × *g* for 10 min and visible buffy coat material aspirated. Contaminating erythrocytes were lysed by addition of 10 ml of Pharmlyse Buffer (BD Biosciences, Oxford, UK) and incubation for 10 min at room temperature (RT) before being washed three times in HBSS (Life Technologies, ThermoFisher Scientific, Paisley, UK). Virus isolation was carried out on fBT cells as described (7).

### Serum Neutralization Tests and BVDV Antibody ELISA

Blood samples without anti-coagulant were collected on 0 and 21 dpi. The samples were left at RT for 2 h to clot prior to centrifugation at 1500 × *g* for 10 min. Samples were tested for BVDV-2 neutralizing antibodies by serum neutralization test, using a heterologous strain, NADL (18). BVDV-specific antibody was detected in serum samples using a commercial indirect ELISA (HerdChek^®^ BVDV Antibody ELISA, IDEXX Laboratories, Wetherby, UK) performed according to the manufacturer’s protocol.

### Hematology

EDTA-treated blood samples for platelet and differential leukocyte counts were collected daily from −2 to 14 dpi and on 16, 18, and 20 dpi. Platelet and leukocytes were enumerated by volumetric flow cytometry. In brief, EDTA blood was centrifuged at 200 × *g* for 1 min and 5 µl of platelet-rich plasma was diluted in 2 ml of CellFIX solution (BD Biosciences). The number of platelets was determined using a volumetric flow cytometer (MACSQuant Analyzer; Miltenyi Biotec, Bisley, UK). For total leukocyte counts, EDTA blood was stained with anti-CD45-FITC mAb clone 1.11.32 (IgG1) (Bio-Rad Antibodies, Oxford, UK) and cell counts were obtained by gating FITC positive events (19).

### RT-PCR Detection of BVDV RNA

EDTA blood samples were taken from group 1A and group 1B calves, that were remaining on 28, 42, 55, 70, and 90 dpi. RNA was extracted from 140 µl of blood using the QIAamp viral RNA extraction kit (Qiagen, Crawley, UK) according to the manufacturer’s procedure. Viral RNA was detected using a one-step short-target real-time RT-PCR assay executed for 50 cycles, which detects both strands of BVDV RNA (20). Selected RT-PCR positive samples were further tested using a two-step negative-strand-specific RT-PCR that only detects the presence of the replicative intermediate evident during BVDV genome replication (7).

### Statistical Analysis

Clinical scores were analyzed by Kruskal–Wallis ranking test. Statistical significance between groups and between pre- and post-challenge levels was determined using a one-way ANOVA test. A Bonferroni adjusted *p*-value of 0.007 was considered indicative of significance.

## Results

**Figure 1:**
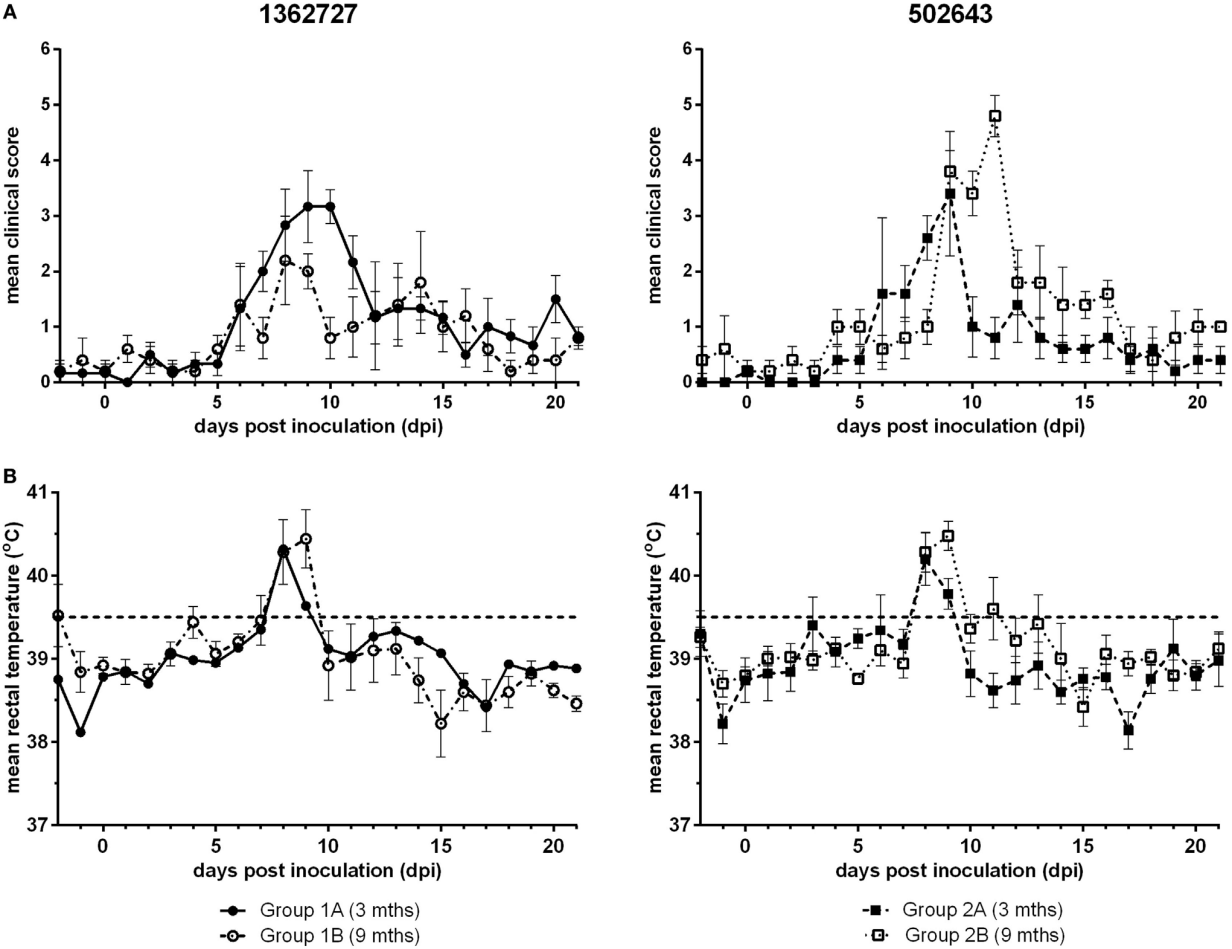
Assessment of the clinical signs and pyrexia induced following experimental infection of calves with BVDV-2 isolates. Mean clinical scores ± SEM **(A)** and rectal temperatures ± SEM **(B)** following inoculation with BVDV-2 isolates 1362727 (Group 1) and 502643 (Group 2) were assessed in 3-month (Groups 1A and 2A) and 9-month-old calves (Groups 1B and 2B).

Calves inoculated with either BVDV-2 strain, 1362727 or 502643, exhibited nasal discharge, coughing, mild depression, and inappetence with a peak in mean clinical score between 8 and 11 dpi (Figure 1A). All calves in all groups developed mild to moderate clinical disease. Group 1A calves (3-month-old calves inoculated with strain 1362727) achieved a higher mean clinical score that was sustained for a longer period compared to the group 1B calves (9 months old; 1362727). By contrast, group 2B calves (9-month-old calves inoculated with strain 502643) reached a higher mean clinical score than group 2A calves (3 months old; 502643). Although, the differences in clinical score observed were not statistically significant (*p* = 0.305). After inoculation with either BVDV-2 strains, all animals independent of age exhibited pyrexia (>39.5^o^C) by 8 dpi with this lasting for 2 days in majority of the animals (Figure 1B).

**Figure 2:**
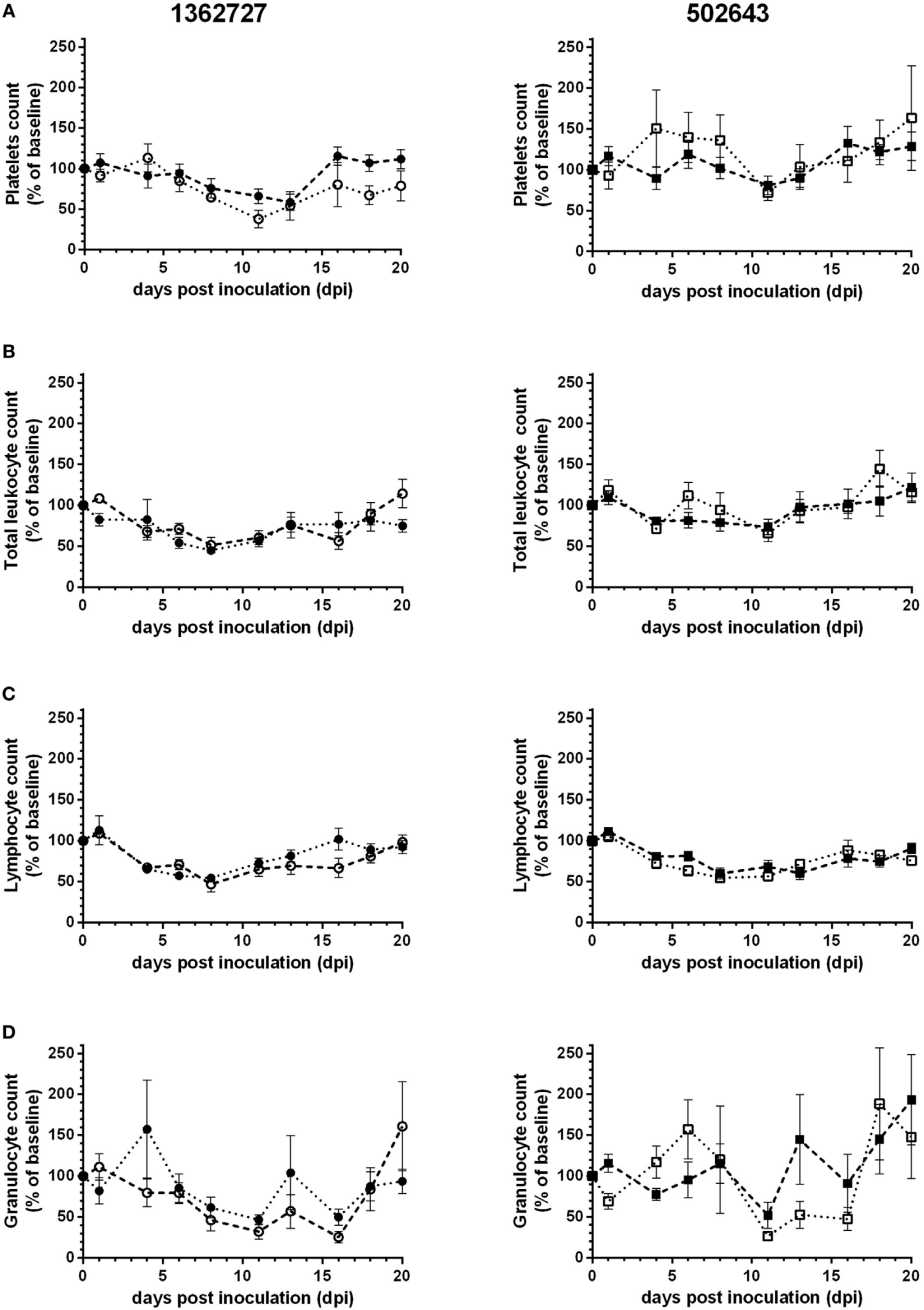

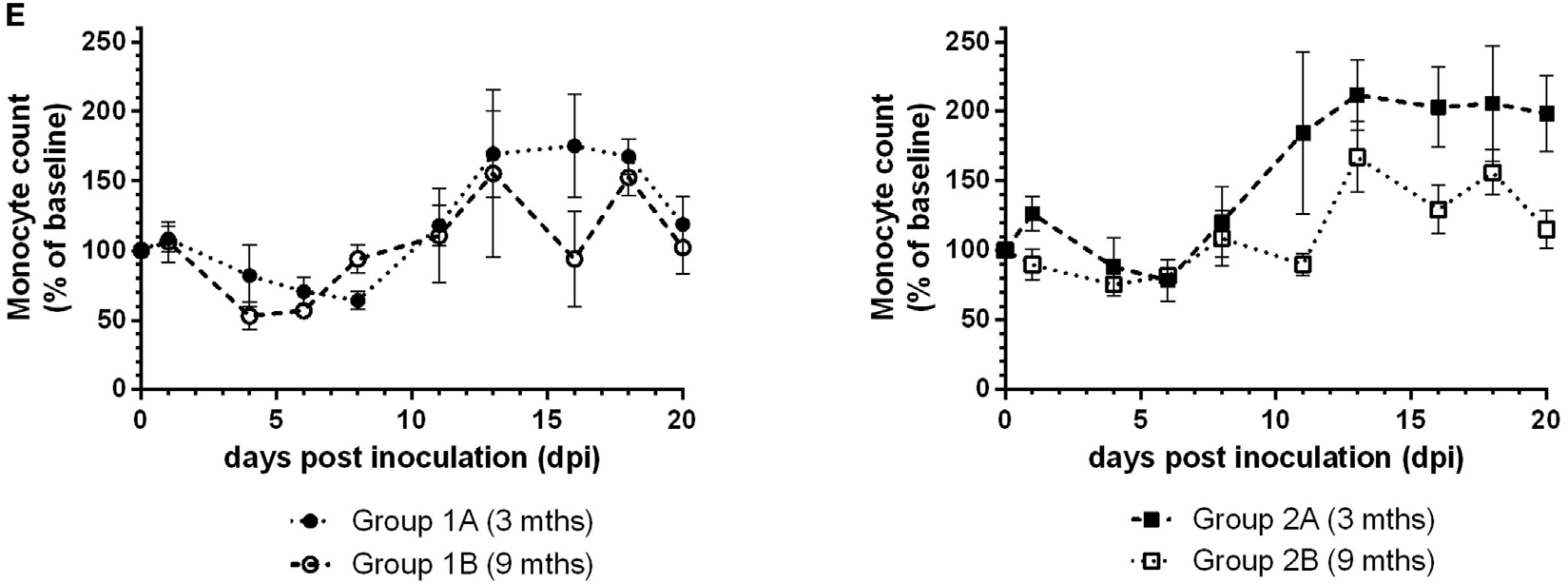
Assessment of hematological parameters following experimental infection of calves with BVDV-2 isolates. Mean values ± SEM for platelets **(A)**, total leukocytes **(B)**, lymphocytes **(C)**, granulocytes **(D)**, and monocyte **(E)** counts are displayed as a percentage of pre-inoculation baseline counts following inoculation with BVDV-2 isolates 1362727 (Group 1) and 502643 (Group 2) in 3-month (Groups 1A and 2A) and 9-month-old calves (Groups 1B and 2B).

Hematology counts were calculated as a percentage of the pre-inoculation baseline. All groups showed a decrease, albeit not statistically significant compared to pre-inoculation levels (*p* = 0.14), in mean platelets count and reached lowest levels between 11 and 13 dpi (Figure 2A). Group 1B animals showed the greatest drop in counts to as low as 38% of the pre-inoculation level at 11 dpi. Platelets count increased toward baseline levels as the study progressed. Based on the mean total leukocyte count, all groups of calves exhibited a transient leukopenia during the study (Figure 2B). Calves inoculated with strain 1362727 showed the more marked response compared to 502643 inoculated calves with levels dropping on 8 dpi to as low as 45 and 52% of the baseline level for groups 1A and 1B, respectively. There was a minimal decrease in mean total leukocyte count for group 2B calves due to only two of the five animals showing any notable decrease in total leukocyte counts. In all groups, mean lymphocyte counts decreased to lowest levels at 8 dpi (Figure 2C) with group 1B animals showing the greatest drop to 47% of pre-inoculation levels, although this was not significant compared to pre-inoculation levels (*p* = 0.15). Granulocyte levels initially dropped to the lowest level at 11 dpi in all groups (Figure 2D). Although a further decrease to 27% of the pre-inoculation baseline was reached for group 1B at 16 dpi. All groups showed monocytosis with highest counts ranging from 156– 212% dependent on the inocula and the age of calves (Figure 2E).

Virus was isolated from nasal swabs and leukocytes isolated from EDTA blood samples (Table 1; Table S1 in Supplementary Material). Virus was isolated from nasal swabs from group 1A calves inoculated with BVDV-2 strain 1362727 from 2 to 14 dpi and for group 1B animals from 4 to 10 dpi and 13 to 14 dpi. For the 502643 strain, virus isolations from nasal swabs were positive 2, 4–10, and 16 dpi for animals in group 2A with isolations from all animals on 5 and 9 dpi and from the group 2B animals from 4 to 10 dpi and 12–13 dpi. Virus was isolated from leukocytes on 3–12, 14, and 16 dpi for the group 1A animals with isolations from all animals on 5, 7, and 8 dpi. For the group 1B calves, virus was isolated on 5–11 and 13 dpi with all animals testing positive on 5 dpi. Virus was isolated on 5–9, 11–12, and 16 dpi for the group 2A calves with isolations from all animals on 5 and 8 dpi. For the group 2B animals, virus was isolated on 3–14 dpi with all animals being positive on 8 dpi.

**Table 1:** Summary of Virus isolation data.

Group	Virus strain	Age (months)	Nasal swabs		Leukocytes	
			Range of detection (days)	Peak detection (days)	Range of detection (days)	Peak detection (days)
1A	BVDV-2 (1362727)	3	2–14	–	3–16	5, 7, 8
1B	BVDV-2 (1362727)	9	4–14	–	5–13	5
2A	BVDV-2 (502643)	3	2–16	5, 9	5–16	5, 8
2B	BVDV-2 (502643)	9	4–13	–	3–14	8

**Figure 3:**
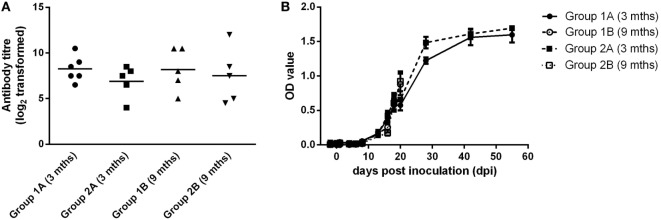
Serological responses following experimental infection of calves with BVDV-2 isolates. Mean serum neutralizing antibody titers (log_2_ transformed) measured at 21 dpi **(A)** and BVDV antibody levels (OD values ± SEM) **(B)** are plotted for calves inoculated with BVDV-2 isolates 1362727 (Group 1) and 502643 (Group 2).

Blood samples were taken from all groups of animals on 0 and 21 dpi. For all groups, calves had seroconverted by 21 dpi (Figure 3A). There was no significant difference between the mean neutralizing antibody titers for any groups. In all groups, BVDV-specific antibody levels increased over the course of the study to levels that were consistent with seroconversion to the virus (Figure 3B).

To investigate the presence of replicating virus after the animals had recovered from an acute infection, group 1B and group 2B animals were maintained up to 90 dpi. Individual EDTA blood samples were tested using the short-target RT-PCR (20) and RNA positive samples were further tested using the negative-strand-specific RT-PCR (7). One of the three calves remaining at 90 dpi was RNA positive when using the short-target PCR which detects both the viral genome and the replicative intermediate. This calf from group 2B also tested positive as late as 70 dpi using the negative-strand-specific RT-PCR which detects only the replicative intermediate (Table 2). Another calf from group 2B was RNA positive at 55 dpi and tested positive using the negative-strand-specific RT-PCR at 42 dpi. Of the three calves from group 1B tested using the short-target RT-PCR, 3 out of 3 were viral RNA positive at 28 dpi and only 1 out of 3 at 42 dpi. These samples were not tested using the negative-strand-specific RT-PCR so it is not clear if there was actively replicating virus at these time-points.

**Table 2:** Ct values for short-target and negative-strand RT-PCR.

Virus	PCR	Days post inoculation				
		28	43	55	70	90
BVDV-2 (502643)	Short target^a^	36.76	40.00	36.01	38.19	40.91
	Negative strand^b^	41.15	38.85	37.61	41.27	No Ct

*^a^One-step real-time RT-PCR detects total viral RNA*.

*^b^Two-step real-time RT-PCR detects only negative-strand viral RNA*.

## Discussion

The purpose of this study was to develop an experimental challenge model for assessing the efficacy of BVDV vaccines using a BVDV-2 strain that would produce measurable outcomes, such as leukopenia and viremia, without severe clinical disease. The second area was to investigate the persistence of BVDV-2 since infections of calves with BVDV-1 resulted in prolonged viremia and replication (6, 7). During the study, mild to moderate clinical symptoms were observed with a peak in clinical symptoms at around 8–10 dpi. However, one animal in group 1A displayed significant nasal discharge at 6 dpi. All the group 2A calves received an anti-inflammatory injection at 11 dpi due to dullness, pyrexia, and nasal discharge. Nevertheless, there was no marked difference in mean clinical score, leukocyte, or platelet counts between this group and the group 2B calves that were infected with the same BVDV strain at the same dose. The requirement for veterinary intervention may be attributed to the younger age of the calves in group 2A compared to 2B. A single group 1B calf required veterinary intervention during the study possibly due to secondary opportunistic bacterial infection that may have already been present subclinically in the animal before the onset of the study. This calf exhibited a prolonged pyrexia, inappetance and nasal discharge, and lower leukocyte and platelet counts compared to others group 1B calves. This calf was treated with antibiotics and anti-inflammatories and clinical signs resolved and leukocyte and platelet counts returned to baseline levels in line with the other calves.

During the study, leukopenia was evident in all groups of calves with associated lymphopenia and neutropenia which is consistent with other experimental intranasal infection with both high and low virulent BVDV-2 isolates with the greater decrease associated with the high-virulence strains (21, 22). Indeed, inoculation with strain 1362727 promoted a greater decrease in total leukocyte counts compared to strain 502643 consistent with the more virulent nature of strain 1362727 compared to strain 502643. All calves in groups 1A and 1B, inoculated with 1362727, developed thrombocytopenia with levels recovering by 3 weeks post inoculation. Little such changes were seen in group 2A and 2B animals, inoculated with 502653, even though these animals showed clear signs of infection. Thrombocytopenia has been associated with experimental infection of calves with either BVDV-1 or -2 isolates (7, 22–25) and in comparative studies of high and low virulent strains thrombocytopenia has not developed in calves inoculated with a low virulent strain (22). The monocytosis associated with BVDV infection has been described previously during a study of naturally BVDV-1 infected calves (26). However, this is in contrast to an experimental intranasal infection with BVDV-2 where a monocytopenia was reported (27).

Virus was isolated from both nasal swabs and peripheral blood leukocytes from each animal regardless of the inoculated strain or age of animals, which is consistent with previous studies of acute BVDV infection (7). Although in this study no naïve “in-contact” animals were included, the presence of virus in the nasal swabs shows the potential for horizontal transmission. The peak of virus isolation from both nasal swab and leukocyte samples coincided with the peak of clinical signs and pyrexia observed in all groups. The presence of BVDV RNA after the clinical signs have resolved and animals have seroconverted has been reported previously (6, 7, 28–30). The presence of the replicative intermediate as late as 70 dpi is consistent with our previous work with experimental BVDV-1 infection (7) and this indicates that the virus is still replicating long after the calves have recovered from the initial clinical infection. It is not clear from this study if these calves were capable of shedding and/or transmitting virus as nasal swabs were not taken at these time points and naïve animals were not present. However, previous studies with a BVDV-1 strain demonstrated viral RNA in nasal swabs as late as 80 dpi. Although, there was no transmission of virus to two naïve calves introduced at 50 dpi and maintained until the end of the study (7). Other experimental infections with BVDV-1 strains have also been unable to demonstrate the ability of these viral RNA positive, antibody-positive calves to transmit virus to BVDV-naïve herd mates (6). Although studies have shown that viral RNA-positive semen and blood samples collected from animals that have recovered from the initial infection can infect naïve cattle when injected intravenously (5, 6). This raises questions about the BVDV-free status of calves that have recovered from acute infections and mounted an antibody response and whether they pose a risk to BVDV-naïve animals. This risk may be lower than that posed by PI calves, which shed large amounts of virus throughout their life. However, these PI calves are identified and removed from herds and these recovered calves may act as “virus carriers,” thus providing a potential source of infection for naïve herd mates in a herd deemed to be BVDV free. Further studies need to be carried out to try to understand the mechanism by which the virus replicates long after the acute infection and the impact that this may have on BVDV-naïve herd mates in the field particularly during BVDV control programs.

The experimental infection reported here for strain 1362727 showed a milder progression of disease in comparison to the severe clinical disease observed in the original field infections (13, 31). Previous studies have proposed that other factors may have an impact on the progression of the disease in the field, such as environmental factors, immunocompetence, nutritional status, and pre-existing infections. This is supported by our previous study, where a 10^5.55^ TCID_50_ dose of a highly pathogenic field strain of BVDV-1 gave rise to a moderate clinical outcome from which the calves would recover rather than succumb as in the original field situation. Interestingly, inoculation with strain 502643 originally isolated from a persistently infected bull with ill-thrift which was present in an asymptomatic herd gave rise to a measurable clinical outcome.

The potential for using either of these viruses as challenge strains for BVDV vaccine efficacy studies requires that there is measurable clinical disease that does not pose problems for animal welfare. This was apparent for strains 1362727 and 502643 as both these strains generated disease with overt clinical symptoms, such as pyrexia, nasal discharge, and inappetence, in all calves in both age groups. Infectious virus was isolated from nasal swabs and leukocyte samples from calves inoculated with either strain independent of the age of the calves. However, a higher number of the calves inoculated with strain 1362727 achieved a higher than 40% decrease in both leukocyte (group 1A—6 out of 6; group 1B—4 out of 5) and platelet counts (group 1A—4 out of 6; group 1B—4 out of 5) compared to those calves inoculated with strain 502643 in which only 2 out of 5 calves demonstrated this decrease. This would suggest that strain 1362727 is an attractive candidate for future efficacy studies as it demonstrated measurable clinical parameters but these were not severe enough to warrant euthanasia of any of the calves.

In conclusion, we present a robust experimental challenge model for BVDV-2 which induces a range of clinical signs, measurable hematological alterations, and viral loads in peripheral blood and secretions. As such these models will aid in the further characterization of BVDV-2 infections and provide a challenge system with which to assess the protective efficacy of existing and novel BVDV vaccines. It can also be used for further host–pathogen interaction studies to resolve the nature of the long-term replication of BVDV in host animals.

## Ethics Statement

The animal study was conducted at Contract Research Unit of the Royal Veterinary College under Home Office License number PPL 70/6459 Protocol 19b5 and approved by the Pfizer Animal Health Animal Welfare Committee.

## Author Contributions

RS was involved in the design of the work, sample collection, data analysis, and interpretation of data for the paper. RS drafted the initial manuscript and revised it critically and gave final approval of the version to be published. SG and SR were involved in the design of the work, sample collection, data analysis, and interpretation of data for the paper. SG and SR were involved in drafting the initial manuscript and revised it critically, and gave final approval of the version to be published. RR, IV, and FS were involved in conception and design of the work, and interpretation of data for the paper. RR, IV, and FS critically revised the work and gave final approval of the version to be published.

## Conflict of Interest Statement

Authors employed at the Animal Health and Plant Agency have no competing interests. Two of the authors (RR and IV) were employed by Pfizer Animal Health (now Zoetis) at the time of the study. Zoetis were involved in the study design and data collection but played no role in the decision to publish or the analysis or interpretation of data.
